# Presenteeism and Productivity: The Role of Biomarkers and Hormones

**DOI:** 10.3390/ijerph18095014

**Published:** 2021-05-10

**Authors:** Aristides I. Ferreira, Amalia R. Pérez-Nebra, Eva Ellen Costa, Maria Luisa A. Aguiar, Adriane Zambonato, Carla G. Costa, João G. Modesto, Paula da Costa Ferreira

**Affiliations:** 1 ISCTE—Instituto Universitário de Lisboa (ISCTE-IUL), Avenida das Forças Armadas, 1649-026 Lisboa, Portugal; 2 UnB—Universidade de Brasília, Campus Universitário Darcy Ribeiro, Brasília, DF 70910-900, Brazil; pereznebra@gmail.com; 3 Department of Psychology, Universidad Internacional de Valencia, Calle Pintor Sorolla, 21, 46002 Valencia, Spain; 4 ISCSP—Instituto Superior de Ciências Sociais e Politicas da Universidade de Lisboa, R. Almerindo Lessa, 1300-666 Lisboa, Portugal; Eva_Ellen_Costa@iscte-iul.pt; 5 UniCEUB—Centro Universitário de Brasília, SEPN 707/907, Campus do UniCEUB, Bloco 9, Asa Norte, Brasília, DF 70910-900, Brazil; malu.aaguiar@gmail.com (M.L.A.A.); adriane.zambonato@uniceub.br (A.Z.); jg.modesto@gmail.com (J.G.M.); 6 ISMAT—Instituto Superior Manuel Teixeira Gomes, R. Dr. Estevão de Vasconcelos 33 A, 8500-656 Portimão, Portugal; carla_filipa_costa@iscte-iul.pt; 7 UEG—Universidade Estadual de Goiás, Av. Universitária, S/N—Nordeste, Formosa, GO 73807-250, Brazil; 8 CICPSI (UIDB/04527/2020; UIDP/04527/2020)—Faculty of Psychology, University of Lisbon, Alameda da Universidade, 1649-013 Lisboa, Portugal; paula.ferreira@campus.ul.pt

**Keywords:** biomarkers, hormones, cortisol, glycemia, presenteeism, productivity

## Abstract

Purpose. This study aimed to assess whether self-reported productivity despite presenteeism may be affected by biomarkers and hormones and how these physiological indicators can interact with each other to explain the presenteeism dimensions. Methods. This pilot study included 180 healthy participants with a mean age of 41.22 years (SD = 13.58), 76.11% of whom were female. The dependent variable included a self-reported measure of productivity loss due to presenteeism: the Stanford Presenteeism Scale 6. This study also includes physiological indicators such as biomarkers (C-reactive protein (CRP) and blood glucose) and hormones (cortisol and TSH thyroid hormone). Results. Multiple linear regression analyses revealed that CRP moderated the relationship between cortisol levels and productivity despite presenteeism. Moreover, the increase of TSH moderated the relationship between cortisol, glycemia, and employees’ capacity to complete work tasks while sick. Conclusions. The results highlight TSH’s moderating role in decreasing employees’ capacity to fulfill tasks when these individuals have high levels of glycemia and cortisol in their blood. These findings have practical and theoretical implications based on a fuller understanding of how biomarkers and hormones explain productivity despite presenteeism.

## 1. Introduction

Presenteeism refers to working while one is sick and can cause significant productivity losses associated with poor health, emotional exhaustion and workplace epidemics from virus transmission such as the current SARS CoV-2 [1]. However, the presenteeism literature has been very critical regarding the measurement of presenteeism with recent studies, suggesting the poor psychometric evidence of the current self-reported measures [2]. Therefore, as mentioned in previous studies, an ideal measure of presenteeism should include both psychological and physical conditions that affect productivity while being ill [2,3]. In fact, there is a call to include physiologic measures of stress (e.g., cortisol) with classical self-reported instruments of presenteeism/productivity losses due to presenteeism. With this in mind, we are strongly convinced that the current study may provide important contributions to evaluate productivity while one is ill and the development of health promotion programs [2]. 

The excessive and accumulative exposure to stressors at work may conduct to increased morbidity (e.g., diabetes, inflammations). In fact, previous research shows that stress activities increase cortisol production [4,5], hyperglycemia [6], and systemic inflammation [7]. Therefore, the present research aimed to investigate how presenteeism may be associated with biological variables by detecting interactions between biomarkers and hormones (e.g., cortisol and TSH) and presenteeism’s effect on job performance. Due to the scarcity of studies [2], we aimed to explore associations between all the analyzed biomarkers and then find possible interactions.

The presenteeism literature has considered two different approaches: (i) the frequency of presenteeism, and (ii) productivity loss associated with presenteeism. In the current study we will consider the latter perspective. To this end, the Stanford Presenteeism Scale 6 (SPS-6) was used to measure productivity despite sickness presence (presenteeism) [8]. This methodological approach considers two dimensions: one related to psychological illness and avoidance distraction, and another related to physical illness and completed work. Presenteeism refers to attendance behavior in the workplace, when employees cannot fulfill their functions adequately due to physical or psychological problems [9,10]. The literature suggests that several health conditions explain productivity despite illness [11]. For example, in a study conducted with a sample of 296 nurses, lower-back pain and breath infections were among the most prevalent physical diseases [8,12]. Regarding psychological diseases, this study also showed that stress and anxiety were the most prevalent health conditions in nurses. 

Cortisol is mostly used as a marker of different stress types [4,5,13,14]. Studies associating perceptions of daily experiences with physiological processes have confirmed relationships between negative experiences, stress, and physiological endeavors involving increased cardiovascular activity [14] and higher cortisol levels [4,14]. Despite its pertinence, studies examining the impact of cortisol levels on job performance considering multicultural perspectives have been scarce in the literature. Previous studies have shown that immunological activity depends on positive mood reactivity and that stress activities increase cortisol production [4,5,15]. Moreover, researchers have found evidence that low levels of cortisol in conjunction with high levels of CRP, a marker of systemic inflammation, influence individuals with depression by increasing their stress sensitivity and negative affect reactivity [7]. Another study revealed that CRP is correlated with disease severity in patients with chronic spontaneous urticaria [16]. However, despite the significant correlations found in previous studies, some inconsistency has been reported in the association between stress and CRP [17]. 

Another significant biomarker in this context is glycemia, which is related to cognitive decline—a process subject to biological constraints [18] and thus, more related to the avoidance distraction dimension of presenteeism. Hyperglycemia is known to cause a gradual decrease in cognitive functions and has been associated with increased absenteeism [19,20] and burnout [6]. Both reflect adverse implications on job outcomes. Moreover, there is also literature suggesting that higher values of CRP and hyperglycemia have been used as blood parameters to detect long-term diseases such as diabetic foot osteomyelitis [21]. These findings provide interesting clues about the possibility that having higher levels of CRP and glycemia could reinforce the detrimental role of cortisol in decreasing workers’ productivity when they go to work while they are ill. In other words, the presence of an interaction effect implies that the negative effect of blood cortisol levels on productivity while one is ill varies as a function of low versus high levels of CRP and glycemia. Taking into account the aforementioned evidence, individuals with high levels of cortisol who are also diagnosed with high CRP and glycemia have a higher tendency to reduce their capacity to concentrate and accomplish assigned tasks at work while they are ill. Hence, the following hypotheses were formulated for the present study:

**Hypotheses 1** **(H1).** *Blood CRP levels will moderate the relationship between blood cortisol levels and productivity while one is ill (presenteeism). The negative relationship between these variables (i.e., cortisol and productivity) will be stronger for workers who have higher CRP and glycemia levels.*

**Hypotheses 2** **(H2).** *Blood glycemia levels will moderate the relationship between blood cortisol levels and productivity while one is ill (presenteeism). The negative relationship between these variables (i.e., cortisol and productivity) will be stronger for workers who have higher CRP and glycemia levels.*

Studies which used a pool of variables have revealed that thyroid-stimulating hormone (TSH) in subclinical hypothyroidism has a role in psychological consequences [21,22]. The thyroid gland produces stimulating hormones that help control and regulate the amount of energy used in the body (i.e., metabolism). The increased amount of TSH stimulates the thyroid gland to produce the hormone thyroxine (T4) and triiodothyronine hormone (T3), which accelerate the metabolism and contribute to symptoms such as irritability, weight loss, an increased heart rate, excessive perspiration, and heat intolerance [23]. Thyroid clinical symptoms have been associated with tiredness, weight gain, excessive sleepiness and physical weakness [24], which may affect productivity at work. Thyroid hormones have an important role in the glucose metabolism [25] with studies suggesting interactions between the hypothalamic–pituitary–adrenal axis and insulin [26]. Thyroid hormone imbalance appears in combination with metabolic disorders, such as diabetes, which may worsen health conditions and affect employees’ productivity. A recent study conducted with a sample of 13,292 participants revealed that a group with high levels of glucose was associated with high presenteeism productivity losses when compared to a group with low blood glucose [27]. Based on these assumptions, we expect that increased levels of both blood glucose and TSH influence sick workers’ ability to complete their tasks. Apparently, not only do high levels of glycemia contribute to the negative affect of cortisol on productivity while individuals are ill (H2), but they also hinder the negative relationship between TSH and employees’ capacity to perform tasks during sickness disease. Therefore, the following hypothesis was postulated for the present study:

**Hypotheses 3** **(H3).** *Individuals with high levels of TSH that interact with high blood glucose will have greater difficulty in presenting higher levels of productivity while they are ill.*

**Figure 1:**
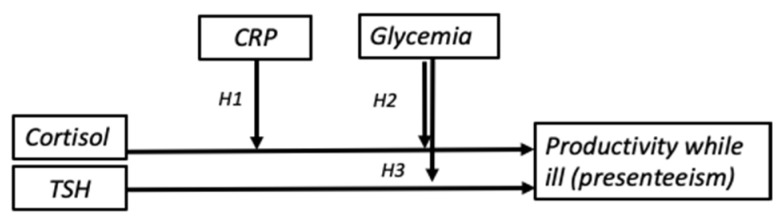
Research model diagram.

Figure 1 shows the proposed relationship amongst the studied variables.

## 2. Methods

### 2.1. Participants and Procedures

The participants were 108 active Brazilian employees who were users of a university laboratory in Brasília (Brazil) and 72 Portuguese employees from a Portuguese company that provides health care services. In total, our sample comprises 180 active employees, of whom 137 were female. Their mean age was 41.22 years (standard deviation [SD] = 13.58). Regarding education and employment, 50.6% of the sample had a high school diploma and 37.1% a university degree. Eighty-four participants (46.7%) were removed from the analysis because they did not report health problems (e.g., anxiety, depression, migraine, arterial hypertension, lower-back pain, allergies, dermatitis…) in the six months prior to this study (which was a requisite for presenteeism, according to the Stanford Presenteeism Scale). Moreover, exclusion criteria included hypothyroidism, hyperthyroidism or diabetes which had already been diagnosed, medicament use to control glycemia, depression, infection, and hormone replacement procedures that could affect medical exams. The final sample was comprised of 97 participants, 60 from Brazil and 37 from Portugal. 

In order to test if higher levels of CRP and glycemia explain a negative relationship between cortisol and workers’ productivity, blood samples were taken from the participants, who then filled out a questionnaire in two locations, one in Brazil and another in Portugal. The Brazilian sample was collected in the Community Treatment Center (CAC) of the University Center located in the Southern Commercial Sector of the city of Brasília, in Brazil’s Federal District. This center usually serves the underserved population, and most of the sample was composed of healthcare employees: attendants, secretaries, caregivers (i.e., housemaids, nannies, caregivers of the elderly, cleaners). Our sample also included plumbers, bricklayers, among others. The CAC is a university laboratory that serves the surrounding community. In this case, the recruitment procedure was the following: we offered participants the opportunity to participate in a research about the relation between blood markers and psychological factors. We provided complementary exams for free when participants did not have prescriptions for them (e.g., cortisol, TSH). We offered this to up to 6 participants per day, due to the number of available slots for the cortisol test.

The Portuguese sample was collected in a company that provides external services in Portugal (Lisbon) in the areas of occupational safety and health (e.g., nurses, doctors and administrative staff). The research was supported by the company’s executive board that accepted to participate in the study and invited all employees (100 in total) to do so as well. Before agreeing to participate, the patients were informed of the study’s objectives, and they were asked to sign an informed consent term. Those who agreed to participate filled out the questionnaire while waiting to be called to give blood samples. Due to financial constraints, we could only count measures of CRP in the Brazilian sample. The questionnaires were answered using paper and pen. 

The blood samples were taken in the morning before the workday, following a 20 min rest, and after participants fasted for 8 h. Three separating gel vacuum tubes were used, except for the full blood count. Each tube held 4 mL of blood and contained the following biological reagents: plasma cortisol, with an obligatory rest of 20 min before the sample was taken; thyroid hormone, fasting glycemia, and full blood count measures of CRP. 

To maintain the confidentiality and anonymity of the data, the laboratory gave us a protocol number. This number was matched with the medical exam results when they arrived by e-mail. 

### 2.2. Instruments

The SPS-6 (Stanford Presenteeism Scale—6) was included in the current study. The presenteeism literature shows that the SPS- 6 is one of the most used instruments to measure productivity despite illness and revealed very good psychometric properties in a previous study developed with Portuguese-speaking samples [28]. The original version of this instrument [29] includes six items that evaluate two factors directly related to presenteeism: completed work and concentration that were computed (mean score) at a higher-level factor named “productivity despite illness (Presenteeism)” [30]. The Portuguese version [28] was adopted. We tested its standard assumptions with normality tests (skewness and kurtosis/error), revealing a normative interval |1.7|. Due to demographic differences (e.g., mean age differences between samples), measurement equivalence across groups was computed [31,32,33] (Table 1), which allowed the analysis with the complete sample, as the instrument demonstrated suitable equivalence across countries. The Cronbach alphas were 0.74 and 0.80, omegas were 0.74 and 0.81, and the model showed adequate fit for a second-order general factor of productivity despite illness (χ^2^ = 14.43; df = 7; χ^2^/df = 2.06; CFI = 0.96; TLI = 0.91; SRMR = 0.06), which presented a general omega reliability score of 0.77 [34]. Only participants who reported illness in the last six months were eligible to answer the items. The demographic data includes age, gender, and education.

### 2.3. Statistical Analysis and Reference Values

The reference values for each biochemical marker were normal CRP levels (below 3.0 mg/l), glycemia (70–99 mg/dl), cortisol (4.3–22.4 g/dl), and TSH (0.55–4.78 mcUI/mL). In most cases, the samples were processed using enzymatic with hexokinase to conduct glycemia test; immunoturbidimetry to test CRP; and electrochemiluminescence to test hormones. Questionnaire and laboratory data were collected gathered on the same day.

We tested normality and homoscedasticity assumptions. CRP and Cortisol data were normally distributed, whereas glycemia and TSH revealed some non-normal parameters. For TSH, we found one potential outlier higher than 2SD (TSH = 75.4), whom we opted to delete from the database. The Levene test was non-significant for all markers.

Before testing the hypotheses, comparative tests between the samples were conducted and, in general, no significant differences were found except for glycemia, cortisol, and level of education. The Brazilian sample revealed higher glycemic levels (M_B_ = 101.90; SD = 26.72; M_P_ = 84.89; SD = 10.38, *p* < 0.05), and lower education levels (77.77% of Brazilian and 40.00% of Portuguese participants had a high school degree or lower). The Portuguese sample revealed higher levels of cortisol (M_B_ = 13.43; SD = 5.82; M_P_ = 17.48; SD = 7.16, *p* < 0.05). Although there were differences between the samples (*p* < 0.05), all were within the standard norms [35]. We used the IBM SPSS Software (IBM, New York, USA) and the PROCESS Macro (University of Calgary, Alberta, Canada) [36]. Therefore, hypotheses 1 and 2 were tested through a multiple moderation analysis where the effect of cortisol could also be a function of two variables simultaneously, such as both CRP and glycemia (Model 2 in PROCESS), while Hypothesis 3 was tested using a simple moderation analysis (Model 1 in PROCESS). All the variables were mean-centered prior to analysis.

### 2.4. Ethical Approval

All procedures performed in this study were in accordance with the institutional and/or national research committee’s ethical standards and with the 1964 Helsinki declaration and its later amendments or comparable ethical standards. Approved in CAAE: 51340715.2.0000.0023.

## 3. Results

Table 2 presents the means, standard deviations, and Pearson’s product–moment correlations among the studied variables with the final sample. 

In Table 3, the results showed evidence of a moderation effect between cortisol and CRP and between cortisol and glycemia in terms of productivity despite illness [F(1,40) = 6.10; *p* = 0.02, and F(1,40) = 5.94; *p* = 0.02, respectively]. It is important to notice that presenteeism is a trick variable because increasing its level is worse for individuals and organizations. Thus, there is a loss in performance when Cortisol, CRP and TSH levels are increased.

**Figure 2:**
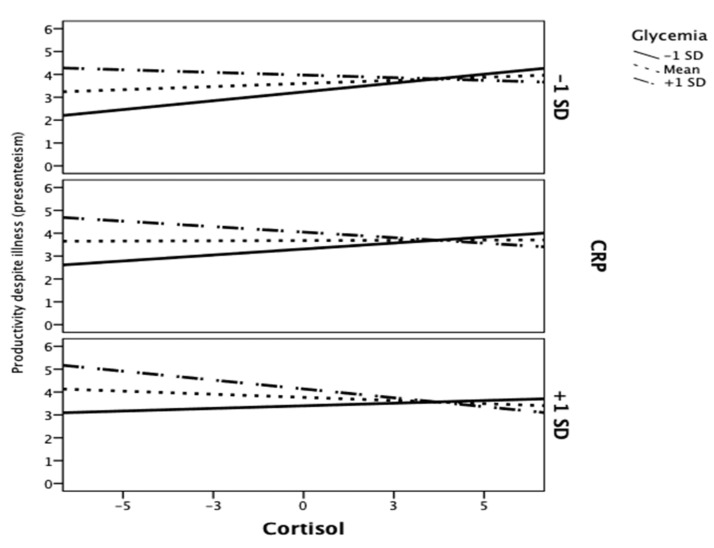
Interaction of Cortisol and CRP and Cortisol and Glycemia levels predicting productivity despite illness (presenteeism).

**Figure 3:**
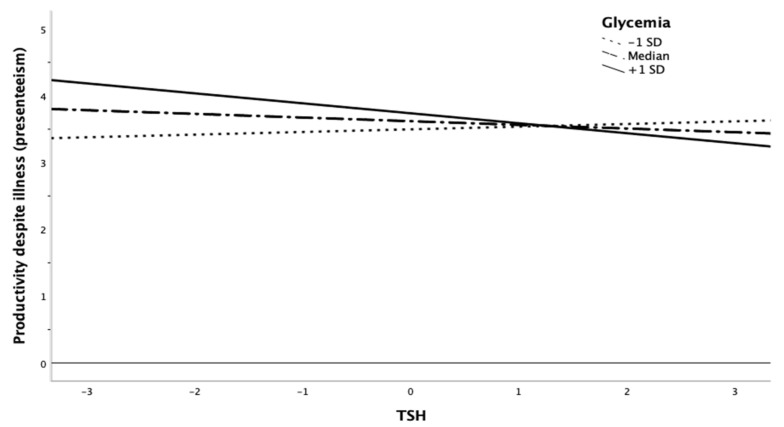
Interaction of TSH and glycemia predicting productivity despite illness (presenteeism).

Our findings showed that, for high levels of CRP (i.e., +1SD > 3.29) and glycemia (i.e., +1SD > 27.09), the relationship between cortisol and productivity despite illness became significantly negative (Figure 2). In addition, our results also revealed (see Figure 3) that employees with high levels of TSH and high glycemia values (i.e., +1SD > 21.54) showed lower levels of productivity despite their illness (F (1,92) = 4.48; *p* = 0.04). 

## 4. Discussion

This study aimed to combine a self-reported measure of productivity despite illness (i.e., the SPS-6) with physiological indicators such as biomarkers and hormones [2,3]. The findings presented confirm the hypotheses and provide initial support for an association between hormones (i.e., cortisol and TSH), biomarkers (i.e., CRP, and blood glucose), and the general SPS-6 dimension of productivity despite illness. 

These findings shed light on the presenteeism and occupational and public health literature that could also be interpreted through blood markers. Also, it can explain some inconsistencies previously detected in the link between stress and CRP. In line with prior studies [17], increased cortisol levels (i.e., the primary stress hormone) were significantly associated with CRP. These results corroborate and extend previous studies [7] by showing that the relationship between cortisol and negative work outcomes is conditioned upon high CRP levels and glycemia. In other words, the current findings suggest that high levels of productivity while employees are sick exist when they develop high levels of cortisol, present signs of systemic inflammations (i.e., high CRP) and high levels of glycemia, which are usually connected to the first stages of stress [37].

Moreover, our results emphasize the TSH role in strengthening the relationship between glycemia and workers’ capacity to develop their work while they are sick. The present findings also contribute to the literature by considering the negative impact that high blood glucose has on individuals’ cognitive functions and job performance [18,38] and by showing that high levels of TSH moderate these effects. Thus, the current results indicated that high TSH may increase the detrimental effects of high glycemia on workers’ capacity to perform their tasks. 

It is reasonable to suppose that TSH alone is associated with poor results of workers’ capacity to perform their tasks, since individuals with high TSH levels tend to generate irritability, fatigue, depression and impaired memory [37]. The interesting point is that the combination of high levels of TSH and glycemia can be problematic for individual performance. In other words, our results show that high TSH and glycemia may explain a detriment on employees’ capacity to perform tasks at work while they are sick.

This study presents various limitations. Therefore, its generalizability is limited and cause–effect paths should be interpreted cautiously. Also, hypothesis 1 was only tested in Brazil for the moderating variable CRP. The research was cross-sectional in nature, and it relied on a small sample, which means replications with more diverse and larger samples (e.g., other countries and patients) are needed. The existing literature has shown that CRP is a sensitive, dynamic molecule that rapidly increases stimulation [39]. Future studies should consider longitudinal analyses to study possible cause–effect inferences and possible non-linear effects between the variables under study. 

Despite these limitations, our findings contribute to the literature by showing that the stress models gain new support because inflammatory effects (i.e., high CRP and glycemia) strengthen the impact of cortisol on productivity losses associated with presenteeism. Our findings support the stress response models that are explained by the hypothalamic–pituitary–adrenal (HPA) axis. Inadequate HPA axis response to stress and inadequate cortisol reactions have been associated with inflammatory disorders, which in turn, affect performance at work. This research focused on the importance of biomarkers and hormones when explaining productivity losses associated with presenteeism. By combining current psychometric instruments with emerging physiologic approaches, the results provide valuable new dimensions in the study of presenteeism and potential improvements in workplace productivity [2]. 

### Practical Implications 

This study also contributes to practice by bringing knowledge from the occupational and health sector to human resource departments. Findings suggest that data from biomarkers and hormones may help decision-makers find strategies to reduce several work demands and increase the required resources to reduce stress (cortisol), inflammations (CRP), sugar consumption (glycemia), or thyroid problems. Specifically, managers should consider reducing long working hours [40], developing practices to increase general well-being, such as physical exercise [41] or providing neurofeedback training [42]. Moreover, in light of our findings, we propose that a tight collaboration between occupational doctors and HR managers may be the best solution to provide employees with personalized solutions based on their needs. For example, the development of digital platforms and behaviour change intervention toolkits could provide individual counseling with regards to food choice and health-related behavior at work and in leisure time. Taking into account that different sources of stress may affect different profiles of employees (e.g., blue vs. white collars; youth vs. senior employees), managers should develop customized interventions to control stress and decrease the levels of cortisol and glycemia. For example, managers should provide annual onsite biometric screenings, health webinars with virtual group lectures in real time, digital workshops with interactive education content. These strategies can be developed periodically (e.g., each semester) so that each employee may find the best strategies and behavior to reduce their health risks [43]. 

## 5. Conclusions

This study also provides an important contribution to the literature as it offers insightful information from the area of occupational and health to human resource departments. Specifically, data from biomarkers and hormones may aid decision-makers in finding strategies to reduce work demands and increase necessary resources to reduce stress (cortisol), inflammations (CRP), sugar consumption (glycemia), or thyroid problems.

In the future, other direct measures could be used as alternatives to traditional models used to evaluate workers’ productivity. This innovative approach approximates existing research on presenteeism in occupational health and medicine, thereby offering a new paradigm in analyses of biomarkers and hormones with significant human resource management implications.

## Figures and Tables

**Table 1:** Equivalence measurement of SPS6 across countries.

	χ^2^ (Diff)	DF (Diff)	CFI (Delta)	RMSEA (Delta)
Configural Invariance	19.34	16	0.98	0.06
Configural loading—metric invariance	(1.35)	(4)	(.01)	(0.03)
Configural intercept	(10.81)	(4)	(0.04)	(0.05)
Configural means	(6.28)	(2)	(0.03)	(0.01)

Note. Brazil *N* = 60; Portugal *N* = 37.

**Table 2:** Means, standard deviations, and Pearson correlations among variables.

Variables	Mean	SD	1	2	3	4
Cortisol	15.04	6.66				
2. CRP	2.72	3.28	−0.20			
3. TSH	3.02	7.71	0.08	−0.02		
4. Glycemia	95.34	23.36	−0.06	0.02	−0.04	
5. Productivity Despite Illness (SPS6)	3.61	0.77	0.21 *	0.17	−0.28 **	0.75 **

Note. * *p* < 0.05, ** *p* < 0.01. SD = Standard Deviation.

**Table 3:** Multiple regression analyses and moderation effects explaining the relationships between both biomarkers and hormones and productivity despite illness.

Direct Effects and Moderations	*b*	SE	*t*	*p*	CI (Lower)	CI (Upper)
Cortisol --> SPS6	0.00	0.03	0.15	0.88	−0.05	0.06
CRP --> SPS6	0.03	0.04	0.73	0.47	−0.05	0.10
Cortisol×CRP --> SPS6	−0.02	0.01	−2.47	0.02	−0.03	−0.00
Glycemia --> SPS6	0.01	0.01	2.42	0.02	0.00	0.02
Cortisol × Glycemia --> SPS6	−0.00	0.00	−2.44	0.02	-0.01	−0.00
Conditional effect for low CRP and Glycemia	0.15	0.04	3.86	<0.01	0.07	0.24
Conditional effect for medium CRP and Glycemia	0.00	0.03	0.15	0.88	−0.05	0.06
Conditional effect for high CRP and Glycemia	−0.16	0.07	−2.17	0.04	−0.30	−0.01
TSH --> SPS6	−0.11	0.07	−1.62	0.11	−0.24	0.02
Glycemia --> SPS6	0.01	0.00	1.50	0.14	−0.00	0.01
TSH × Glycemia--> SPS6	−0.01	0.00	−2.11	0.04	−0.02	−0.00
Conditional effect for low Glycemia	0.08	0.11	0.74	0.46	−0.13	0.29
Conditional effect for medium Glycemia	−0.11	0.07	−1.61	0.11	−0.24	0.03
Conditional effect for high Glycemia	−0.30	0.12	−2.57	0.01	−0.53	−0.07

Notes. SPS6—Productivity despite illness. *B*—unstandardized beta values; SE = Standard Errors; *t* = *t*-value; *p* = *p* value; CI = Confidence Intervals.

## Data Availability

Data can be made available upon request.
